# Stages of change for physical activity in adults from Southern Brazil: a population-based survey

**DOI:** 10.1186/1479-5868-4-25

**Published:** 2007-06-08

**Authors:** Samuel C Dumith, Denise P Gigante, Marlos R Domingues

**Affiliations:** 1 Post-Graduate Program in Epidemiology, Federal University of Pelotas, Pelotas, RS, Brazil; 2 Master Program of Population Health, Universidade do Vale do Rio dos Sinos, São Leopoldo, RS, Brazil

## Abstract

**Background:**

There is evidence that physical activity (PA) interventions tailored to individual's stages of change (SoC) are more effective in promote behavior change than "one-size-fits-all" interventions. However, only a few researches have investigated these stages towards PA behavior in representative samples of the population. Thus, the purpose of this study was to determine the prevalence and factors associated with the SoC for PA in a probabilistic sample of adults aged 20 years or over.

**Methods:**

A population-based survey was undertaken in Pelotas, Southern Brazil, in 2005. An algorithm was applied to evaluate the SoC for PA, and PA was defined as being engaged in moderate-to-vigorous PA for at least 20 minutes on three times per week. The covariates collected in the questionnaire were: sex, age, skin color, marital status, education level, economic status, family income, smoking, body mass index (BMI) and self-reported health status. Data analyses were performed through Poisson and multinomial regression, taking the sampling design into account.

**Results:**

Face-to-face interviews were applied to 3136 individuals, corresponding to a response rate of 93.5%. The prevalence across the stages was as follows: 38.3% in precontemplation, 13.0% in contemplation, 19.5% in preparation, 5.2% in action and 24.0% in maintenance. The elderly, married, smokers, and those with lower socioeconomic status were less likely to adopt, initiate and maintain regular PA.

**Conclusion:**

Despite the all benefits of PA, a high proportion of adults from Southern Brazil are physically inactive and do not present intention to engage in regular PA. The profile of those who are inactive but intend to do PA resembles those who are physically active. The findings of the present study can contribute to improve health behaviors and to plan health promotion strategies aimed at increasing the level of PA in the community.

## Background

The effects of physical activity (PA) on health and disease are well established in the literature [1]. Despite the evidence, a large proportion of the population remains sedentary. For example, in the United States (US), 25% of adults are not active at all [1]. In Brazil, only 13% of adults reported being engaged in PA in their leisure time for at least 30 minutes in one or more days per week [2]. Therefore, any effort to increase PA levels could have a large potential impact on public health.

However, PA is a complex behavior, and the incorporation of this habit cannot be summarized as an all-or-none phenomenon [3]. When attempting to design interventions, it is important to realize that the majority of the population is physically inactive and unmotivated to engage in PA. Interventions that target individuals who are ready to start a PA program are unlikely to reach large segments of the population [4]. Otherwise, some randomized controlled trials indicate that tailoring interventions to individuals' stage of change is more effective than "one-size-fits-all" interventions in promoting behavior change [5-7]. Furthermore, a systematic review of the literature published in 2006 provides support for the use of stage-matched PA interventions [8].

The stages of change (SoC), also known as stages of readiness, are the central construct of the transtheoretical model, encompassing behavior, intentional and temporal aspects of behavior change [9]. This model was introduced in the 80's with smoking cessation, and later it was expanded to several health-related behaviors, including PA. It postulates that behavior change has a dynamic nature, and that the individuals move through a series of stages in their attempts to adopt the desired behavior [10].

Assessment of the individual's SoC can provide important additional information about population receptiveness for specific health promotion strategies, and can be inexpensively and easily incorporated into health surveys. Understanding the distribution of the individuals across the SoC enables stage-matched interventions to be developed for the entire population, not only for those ready to change [11]. Nevertheless, very few researchers have investigated the prevalence and factors associated with these stages in population-based samples, especially in developing countries. In Brazil, only one study evaluated the SoC for PA, but it was undertaken among adolescents from private schools [12].

The aim of the present study was to identify the prevalence of the SoC for PA and to investigate the factors associated with each stage in a representative sample of adults from a Southern Brazilian city. In this specific population, previous studies found that 41% of adults were physically inactive [13]; women, the elderly and those with lower socioeconomic status were more likely to perceive barriers to do PA [14]; and most individuals recognize that PA is important for health [15]. The readiness for PA, however, has not been evaluated.

## Methods

A population-based cross-sectional study was carried out between October and December 2005. Adults aged 20 years or over of the urban area of Pelotas, a medium-sized city in Southern Brazil with approximately 320,000 urban inhabitants, were interviewed. Institutionalized individuals (living in asylums, hospitals and prisons) were excluded, as well as those unable to respond the questionnaire due to physical or mental disabilities. The study protocol was submitted and approved by the Ethics Committee of Federal University of Pelotas Medical School. A written informed consent was obtained from the study participants prior to the interview.

The sample was selected in multiple stages. First, all census tracts of the city were ordered according to monthly income of the family head [16], and 119 were randomly selected with proportionate-to-size probability. All households from these sampled census tracts were listed and systematically selected, taking into account the size of the tract (number of households). It resulted, on average, in 13 households sampled in each census tract. All residents aged 20 years or over were interviewed, and those who refused to answer the questionnaire or who were not found after at least three visits to their households, in different days and hours, were considered as non-respondents.

All variables were collected by face-to-face interviews using a standardized questionnaire. The interviewers were women, aged 18 years or older, with high school education, and were blinded to the aims of the study. They were trained for 40 hours through simulated interviews, household approaching techniques discussion, reading and explanation of the questionnaires instructions and role-play exercises. A pilot-study, in a census tract not sampled for the study, was conducted to check the interviewers' performance and to make final adjustments in the instrument. The quality control of data collection comprised a shorter version of the questionnaire, applied by fieldwork supervisors to 10% of the sample, randomly assigned.

The outcomes consisted in the SoC for PA: precontemplation (not engaged in regular PA, and not intending to do so within the next six months); contemplation (not engaged in regular PA, but intending to do so within the next six months); preparation (not engaged in regular PA, but intending to do so within the next 30 days); action (engaged in regular PA, but for less than six months); and maintenance (engaged in regular PA for the past six months or more). The first three stages can be named pre-adoption, and the last two, post-adoption stages [9].

The criterion to define regular PA was to perform moderate-to-vigorous PA on three or more days per week, for at least 20 consecutive minutes each day. Only intentional physical activities, such as recreational, sports and fitness activities were considered. Several tests evaluated the instrument before the beginning of data collection. Additional file 1 contains the instrument applied in this study to measure the SoC for PA.

The questionnaire included the following covariates: sex (male/female); skin color (observed by the interviewer, and classified as white or non-white); age (collected in complete years, and further grouped); marital status (living with or without a partner); education level (years of education, grouped into five groups); economic status (A, B, C, D or E), according to the Criterion of Brazilian Economic Classification [17], which considers household assets, presence of domestic servants and education level of the family head; monthly family income, collected in "Reais" (R$) – R$ 1.00 was equivalent to US$ 0.44 in the data collection period – and divided into quintiles; smoking (non-smoker; former smoker or current smoker); body mass index (BMI), calculated through self-reported weight and height (in kg/m^2^) and categorized into four groups; and self-reported health status (excellent, very good, good, regular or poor).

Since this study was developed within a larger research on health, the sample comprised 3353 adults. The sample size obtained allowed a statistical power of 80%, with a confidence level of 95%, and precision of 1.5 percent points to detect an outcome prevalence of at least 4.0%, with a design effect of 2.0 and an increment of 10% for non-respondents. For testing of associations, the study allowed a power greater than 80% to detect associations for an outcome prevalence of at least 10%, prevalence ratio of 2.0, exposure frequency between 20 and 80%, with a design effect of 2.0 and an increment of 10% for non-respondents.

Double entry and data validation were conducted with automatic checking of amplitude and consistency by *EpiInfo *– version 6.04, and data analyses were undertaken with *Stata Statistical Software *– version 9.2. Following descriptive analyses, associations between the SoC for PA and covariates were tested through Poisson regression [18]. Crude and adjusted analyses were also conducted through multinomial regression, considering the precontemplation stage as the reference category. This statistical regression technique is proper when the outcome is polythomous, and informs the probability to be in a determinate stage according to each independent variable. In order to adjust the effect of each independent variable for possible confounders, a hierarchical model with four levels was elaborated for the multivariable analysis, and the covariates were controlled for those in the same level or in the ones above with a p-value ≤ 0.20 [19]. In the first level, demographic variables (sex, skin color, age and marital status) were included; in the second level, socioeconomic variables (educational level, economic level and family income) were entered; third level included behavioral variables (smoking and BMI); and in the last level, self-reported health status was entered. All analysis took into account the design effect, defining the census tracts as the primary sampling units. All tests were two-tailed, and the significance level was 5%.

## Results

Of the 3353 individuals included in the study, 3136 were interviewed, corresponding to a response rate of 93.5%. The design effect of PA was 1.9, with an intraclass correlation coefficient of 0.035.

Most individuals were women (56%), white (84%), and lived with a partner (63%). Almost 20% were older than 60 years, 25% had four years or less of education, 29% belonged to the lower economic status ("D" or "E"), 27% were current smokers, 16% were obese (BMI ≥ 30 kg/m^2^) and 28% perceived their own health as regular or poor. The mean age was 44 years (SD 16; range 20–104), and the mean family income was US$ 721.00 (SD 1,086.00; median 444.00).

**Figure 1:**
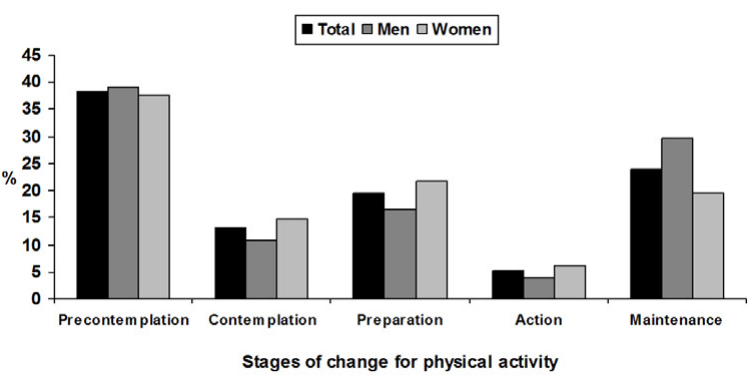
Prevalence of the stages of change for physical activity in the overall sample and stratified by sex. Pelotas, Brazil, 2005.

The distribution of individuals across the SoC for PA (Figure 1) indicates that a large proportion of individuals are inactive and do not intend to engage in PA. Among those who were not regularly active (n = 2220), less than half (45.9%) plans to begin PA, and only about one quarter (27.5%) intents to do so in the near future (next 30 days). Among those who reported to be physically active (n = 916), almost 20% engaged in PA in the last six months.

Table 1 presents the distribution of the SoC for PA according to the covariates. The probability of being in the precontemplation stage did not differ by sex, and was higher for non-white individuals, those living with a partner and for current smokers, compared to their counterparts. Moreover, this probability increased with age, and decreased with education level, economic status, family income, BMI and self-reported health status.

**Table 1:** Sample distribution and prevalence of the stages of change for physical activity according to the covariates^§^. Pelotas, Brazil, 2005.

**Variable**	**N**	**Precontemplation (%)**	**Contemplation (%)**	**Preparation (%)**	**Action (%)**	**Maintenance (%)**
Sex		p = 0.5*	p = 0.002*	p = 0.001*	p = 0.004*	p < 0.001*
Male	1378	39.0	10.8	16.6	3.9	29.7
Female	1758	37.7	14.8	21.7	6.2	19.6
Skin color		p = 0.03*	p = 0.5*	p = 0.4*	p = 0.3*	p = 0.6*
White	2634	37.3	13.3	19.7	5.4	24.3
Non-white	502	43.2	11.8	17.9	4.2	22.9
Age (years)		p < 0.001**	p < 0.001**	p = 0.01**	p < 0.001**	p = 0.9**
20–29	747	27.2	16.1	20.6	8.0	28.1
30–39	611	38.6	15.9	19.2	6.7	19.6
40–49	661	41.6	11.2	23.8	3.9	19.5
50–59	520	39.4	12.3	17.3	3.1	27.9
60–69	342	40.9	12.9	16.1	3.5	26.6
≥ 70	255	55.7	3.9	14.5	2.8	23.1
Marital status		p = 0.002*	p = 0.2*	p = 0.7*	p = 0.2*	p = 0.05*
Without a partner	1168	34.4	13.9	19.8	5.9	26.0
With a partner	1968	40.6	12.5	19.3	4.7	22.9
Education level (years)		p < 0.001**	p < 0.001**	p < 0.001**	p < 0.001**	p < 0.001**
0	209	59.3	5.7	16.3	1.9	16.8
1–4	574	53.1	11.7	12.4	2.8	20.0
5–8	1005	45.7	12.1	18.4	4.7	19.1
9–11	824	26.8	15.8	21.6	6.7	29.1
≥ 12	522	17.4	14.9	27.2	7.7	32.8
Economic status^†^		p < 0.001**	p = 0.1**	p < 0.001**	p < 0.001**	p < 0.001**
A (wealthiest)	161	15.5	14.3	23.6	9.9	36.7
B	824	26.2	14.4	24.2	7.4	27.8
C	1295	41.3	12.9	18.3	4.5	23.0
D	762	50.4	11.4	16.2	3.0	19.0
E	73	47.9	12.3	11.0	4.1	24.7
Family income (quintiles)		p < 0.001**	p = 0.04**	p = 0.007**	p = 0.02**	p < 0.001**
1 (lower)	577	48.0	10.2	16.5	5.2	20.1
2	621	47.8	11.4	18.7	3.4	18.7
3	676	38.8	15.4	17.6	3.8	24.4
4	581	34.9	13.3	20.7	6.0	25.1
5	660	23.7	14.2	23.5	7.4	31.2
Smoking		p < 0.001*	p = 0.3*	p = 0.05*	p = 0.09*	p < 0.001*
Non-smoker	1634	33.8	13.5	20.6	6.1	26.0
Former smoker	664	36.9	11.3	19.9	3.9	28.0
Current smoker	838	48.1	13.5	16.8	4.4	17.2
BMI (kg/m^2^)^‡^		p = 0.001**	p = 0.08**	p < 0.001**	p = 0.7**	p = 0.3**
< 18.5	75	62.6	14.7	6.7	2.7	13.3
18.5 a 24.9	1393	39.3	11.9	17.7	5.5	25.6
25.0 a 29.9	1092	34.6	13.8	21.4	4.7	25.5
≥ 30.0	471	34.4	15.1	24.6	5.9	20.0
Self-reported health status		p < 0.001**	p = 0.2**	p = 0.4**	p = 0.02**	p < 0.001**
Excellent	412	27.7	11.4	19.9	4.8	36.2
Very good	419	27.0	13.1	20.3	9.1	30.5
Good	1428	40.3	13.0	19.5	4.7	22.5
Regular	723	44.0	13.5	19.5	4.7	18.3
Poor	154	52.6	14.9	15.6	2.0	14.9

**Total**	**3136**	**38.3**	**13.0**	**19.5**	**5.2**	**24.0**

^§ ^Percent values in the rows must add 100%.

^† ^Criterion of Brazilian Economic Classification [17].

^‡ ^BMI had 3.3% of missing values; the other variables had less than 1%.

* Wald test for heterogeneity.

** Wald test for linear trend.

The likelihood to adopt PA (individuals in the contemplation or preparation stage) was higher for women, increased with education level, economic status and family income, and decreased with age. Skin color, marital status and self-reported health status were not associated with these stages. Prevalence of the preparation, but not of the contemplation stage, was lower for current smokers, compared to non-smokers and former smokers, and a positive association with BMI was observed.

The probability to initiate (action stage) or to maintain (maintenance stage) PA increased with education level, economic status, family income and self-reported health status. Prevalence of the action stage was higher for women, and decreased with age. In the maintenance stage, men and non-smokers or former smokers were more frequent than women and current smokers, respectively. Skin color, marital status and BMI were not associated with the post-adoption stages.

Considering the crude multinomial analysis (Table 2), women presented a higher probability to be in the contemplation, preparation and action stage, compared to the precontemplation stage. On the other hand, men were more likely to be in the maintenance stage. Skin color was not statistically associated with the SoC for PA, although the prevalence ratios were higher for white subjects. Age was inversely associated with the SoC for PA, that is, the younger and middle aged adults were more likely to be in a further stage compared to the precontemplation stage. The same finding was observed for individuals without a partner, with exception for the preparation stage that was not associated with marital status. Education level, economic status and family income showed a similar trend, with a positive association across all the stages, compared to the reference stage. Non-smokers were more likely than smokers to be in a stage beyond the precontemplation; former smokers present a higher probability to be in the preparation and in the maintenance stage than smokers. The probability to be in the contemplation and in the preparation stage was higher among obese individuals (BMI > 30 kg/m^2^), compared to individuals with normal BMI (< 25 kg/m^2^). No associations were found between BMI and action or maintenance stages. Finally, those individuals who perceived their health as excellent or very good were more likely to be in a further SoC for PA, compared to those that perceived their health as regular or poor.

**Table 2:** Crude multinomial analysis of the stages of change for physical activity according to the covariates^§^. Pelotas, Brazil, 2005.

**Variable**	**N**	**Contemplation PR (95%CI)**	**Preparation PR (95%CI)**	**Action PR (95%CI)**	**Maintenance PR (95%CI)**
Sex		p = 0.002*	p = 0.005*	p = 0.005*	p < 0.001*
Male	1378	0.71 (0.57–0.88)	0.54 (0.60–0.91)	0.60 (0.42–0.86)	1.46 (1.23–1.74)
Female	1758	1.00	1.00	1.00	1.00
Skin color		p = 0.176*	p = 0.120*	p = 0.135*	p = 0.172*
White	2634	1.31 (0.89–1.93)	1.27 (0.91–4.73)	1.48 (0.882.48)	1.23 (0.91–1.64)
Non-white	502	1.00	1.00	1.00	1.00
Age (years)		p < 0.001**	p < 0.001**	p < 0.001**	p = 0.011**
20–39	1358	2.58 (1.75–3.81)	1.89 (1.39–2.58)	3.42 (1.95–5.99)	1.41 (1.06–1.88)
40–59	1181	1.50 (1.03–2.18)	1.58 (1.18–2.11)	1.30 (0.71–2.37)	1.07 (0.82–1.40)
≥ 60	597	1.00	1.00	1.00	1.00
Marital status		p = 0.008*	p = 0.070*	p = 0.027*	p = 0.007*
Without a partner	1168	1.30 (1.07–1.58)	1.21 (0.98–1.49)	1.48 (1.05–2.08)	1.34 (1.09–1.66)
With a partner	1968	1.00	1.00	1.00	1.00
Education level (years)		p < 0.001**	p < 0.001**	p < 0.001**	p < 0.001**
0–4	783	1.00	1.00	1.00	1.00
5–8	1005	1.44 (1.08–1.94)	1.65 (1.23–2.21)	2.20 (1.16–4.18)	1.20 (0.93–1.54)
9–11	824	3.19 (2.28–4.48)	3.29 (2.38–4.56)	5.34 (2.90–9.84)	3.11 (2.30–4.19)
≥ 12	522	4.66 (3.04–7.12)	6.38 (4.32–9.41)	9.43 (4.68–18.98)	5.37 (3.62–7.97)
Economic status^†^		p < 0.001**	p < 0.001**	p < 0.001**	p < 0.001**
A/B (wealthiest)	985	2.57 (1.83–3.62)	3.15 (2.29–4.32)	5.15 (3.21–8.26)	3.07 (2.25–4.19)
C	1295	1.36 (0.99–1.88)	1.42 (1.07–1.88)	1.75 (1.13–2.71)	1.43 (1.12–1.84)
D/E	835	1.00	1.00	1.00	1.00
Family income (quintiles)		p < 0.001**	p < 0.001**	p < 0.001**	p < 0.001**
1 (lower)	577	1.00	1.00	1.00	1.00
2	621	1.12 (0.76–1.65)	1.14 (0.83–1.56)	0.65 (0.36–1.19)	0.93 (0.67–1.29)
3	676	1.86 (1.31–2.66)	1.32 (0.94–1.87)	0.92 (0.52–1.62)	1.50 (1.07–2.12)
4	581	1.78 (1.17–2.70)	1.72 (1.18–2.51)	1.59 (0.91–2.80)	1.72 (1.22–2.41)
5	660	2.83 (1.79–4.47)	2.90 (1.97–4.26)	2.90 (1.64–5.13)	3.15 (2.21–4.49)
Smoking		p = 0.017*	p < 0.001*	p = 0.004*	p < 0.001*
Non-smoker	1634	1.43 (1.09–1.86)	1.74 (1.38–2.20)	1.95 (1.28–2.97)	2.15 (1.66–2.78)
Former smoker	664	1.09 (0.76–1.57)	1.54 (1.14–2.09)	1.16 (0.66–2.02)	2.13 (1.58–2.86)
Current smoker	838	1.00	1.00	1.00	1.00
BMI (kg/m^2^)^‡^		p = 0.011**	p < 0.001**	p = 0.332**	p = 0.734**
< 25.0	1468	0.68 (0.48–0.96)	0.59 (0.43–0.81)	0.76 (0.46–1.25)	1.06 (0.76–1.47)
25.0 a 29.9	1092	0.91 (0.65–1.28)	0.87(0.65–1.15)	0.78 (0.47–1.29)	1.27 (0.92–1.75)
≥ 30.0	471	1.00	1.00	1.00	1.00
Self-reported health status		p = 0.017**	p < 0.001**	p < 0.001**	p < 0.001**
Excellent/very good	831	1.48 (1.10–2.00)	1.78 (1.31–2.14)	2.76 (1.76–4.32)	3.14 (2.32–4.25)
Good	1428	1.07 (0.79–1.43)	1.17 (0.89–1.54)	1.26 (0.78–2.04)	1.44 (1.11–1.87)
Regular/poor	877	1.00	1.00	1.00	1.00

^§ ^Precontemplation stage is the base-outcome.

^† ^Criterion of Brazilian Economic Classification [17].

^‡ ^BMI had 3.3% of missing values; the other variables had less than 1%.

* Wald test for heterogeneity.

** Wald test for linear trend.

When the covariates were analyzed in a multivariable model (Table 3), small changes occurred. Concerning economic status, its effect on contemplation and maintenance stages was no longer significant. The same finding was observed between family income and the preparation and action stages. This failing effect can be explained by the colinearity among these two socioeconomic variables. In the adjusted analysis, smoking remains associated only with the preparation and the maintenance stages. Another change is related to the positive association between BMI and the action stage that was not observed in the crude analysis. Self-reported health status remains positively associated only with the maintenance stage after controlling for the other variables in the adjusted model.

**Table 3:** Adjusted multinomial analysis of the stages of change for physical activity according to the covariates^§^. Pelotas, Brazil, 2005.

**Level**	**Variable**	**Contemplation PR (95%CI)**	**Preparation PR (95%CI)**	**Action PR (95%CI)**	**Maintenance PR (95%CI)**
1	Sex	p = 0.001*	p = 0.006*	p = 0.006*	p < 0.001*
	Male	0.69 (0.55–0.86)	0.75 (0.61–0.92)	0.60 (0.42–0.86)	1.51 (1.27–1.80)
	Female	1.00	1.00	1.00	1.00
1	Skin color	p = 0.121*	p = 0.077*	p = 0.056*	p = 0.105*
	White	1.38 (0.92–2.06)	1.33 (0.97–1.83)	1.68 (0.99–1.85)	1.28 (0.95–1.72)
	Non-white	1.00	1.00	1.00	1.00
1	Age (years)	p < 0.001**	p < 0.001**	p < 0.001**	p = 0.016**
	20–39	2.73 (1.84–4.05)	2.01 (1.47–2.76)	3.89 (2.14–7.09)	1.39 (1.05–1.85)
	40–59	1.58 (1.08–2.32)	1.68 (1.25–2.26)	1.45 (0.78–2.70)	1.13 (0.87–1.47)
	≥ 60	1.00	1.00	1.00	1.00
1	Marital status	p = 0.033*	p = 0.060*	p = 0.018*	p = 0.001*
	Without a partner	1.24 (1.02–1.51)	1.23 (0.99–1.52)	1.53 (1.08–2.17)	1.45 (1.17–1.78)
	With a partner	1.00	1.00	1.00	1.00
2	Education level (years)	p < 0.001**	p < 0.001**	p < 0.001**	p < 0.001**
	0–4	1.00	1.00	1.00	1.00
	5–8	1.13 (0.83–1.53)	1.47 (1.08–2.01)	1.51 (0.78–2.89)	1.13 (0.86–1.48)
	9–11	1.99 (1.39–2.86)	2.54 (1.73–3.71)	2.23 (1.17–4.23)	2.61 (1.89–3.61)
	≥ 12	2.72 (1.68–4.41)	4.02 (2.58–6.26)	3.30 (1.59–6.84)	3.99 (2.61–6.10)
2	Economic status^†^	p = 0.248**	p = 0.004**	p < 0.001**	p = 0.246**
	A/B (wealthiest)	1.35 (0.85–2.15)	1.71 (1.19–2.46)	3.14 (1.79–5.51)	1.29 (0.85–1.96)
	C	0.96 (0.65–1.42)	1.06 (0.78–1.44)	1.32 (0.80–2.18)	1.03 (0.77–1.39)
	D/E	1.00	1.00	1.00	1.00
2	Family income (quintiles)	p = 0.004**	p = 0.305**	p = 0.949**	p < 0.001**
	1 (lower)	1.00	1.00	1.00	1.00
	2	1.17 (0.80–1.72)	1.21 (0.89–1.65)	0.60 (0.32–1.12)	1.02 (0.73–1.40)
	3	1.84 (1.28–2.64)	1.19 (0.83–1.71)	0.69 (0.35–1.36)	1.44 (1.01–2.05)
	4	1.54 (1.00–2.38)	1.18 (0.78–1.78)	0.75 (0.38–1.48)	1.39 (0.97–1.99)
	5	1.97 (1.21–3.23)	1.31 (0.983–2.07)	0.94 (0.43–2.05)	1.84 (1.29–2.64)
3	Smoking	p = 0.760*	p = 0.034*	p = 0.449*	p < 0.001*
	Non-smoker	1.09 (0.81–1.47)	1.32 (1.01–1.71)	1.36 (0.84–2.20)	1.68 (1.28–2.20)
	Former smoker	1.15 (0.78–1.70)	1.51 (1.10–2.09)	1.26 (0.68–2.33)	1.89 (1.38–2.59)
	Current smoker	1.00	1.00	1.00	1.00
3	BMI (kg/m^2^)^‡^	p < 0.001**	p < 0.001**	p = 0.039**	p = 0.396**
	< 25.0	0.54 (0.37–0.79)	0.53 (0.37–0.76)	0.52 (0.29–0.95)	0.93 (0.66–1.32)
	25.0 a 29.9	0.85 (0.59–1.23)	0.78 (0.57–1.07)	0.65 (0.37–1.12)	1.11 (0.79–1.56)
	≥ 30.0	1.00	1.00	1.00	1.00
4	Self-reported health status	p = 0.089**	p = 0.923**	p = 0.679**	p = 0.001**
	Excellent/very good	0.76 (0.54–1.06)	1.02 (0.73–1.44)	1.08 (0.66–1.75)	1.71 (1.25–2.34)
	Good	0.72 (0.53–0.98)	0.91 (0.68–1.23)	0.83 (0.51–1.36)	1.13 (0.88–1.45)
	Regular/poor	1.00	1.00	1.00	1.00

^§ ^Precontemplation stage is the base-outcome.

^† ^Criterion of Brazilian Economic Classification [17].

^‡ ^BMI had 3.3% of missing values; the other variables had less than 1%.

* Wald test for heterogeneity.

** Wald test for linear trend.

## Discussion

The present study investigated the prevalence and factors associated with the SoC for PA in a representative sample of adults from Southern Brazil. Results showed that 38.3% were in precontemplation, 13.0% were in contemplation, 19.5% were in preparation, 5.2% were in action and 24.0% were in maintenance.

Some strengths of the study can be listed. First, to the authors' knowledge, this is the first population-based study to investigate the SoC for PA and associated factors in Brazilian adults. Most studies on this issue were conducted in developed countries and with specific populations [20]. Second, the study had a high response rate (93.5%), which assures a good estimative of the data distribution in the population. Third, since the effect of sampling design was the same as estimated a priori, it is unlikely to have affected the study precision. Fourth, the interviewers were extensively trained for the questionnaires application and were not aware of the purposes and hypothesis of the study. Finally, PA was defined in terms of frequency, duration and intensity, as recommended by Reed et al. [21].

On the other hand, some limitations may have affected the results of this study. One of them lies in the validation of the instrument used, which was translated and adapted to the population context. Nevertheless, there are reasons to believe that this stage algorithm is valid, because significant differences were found between individuals classified into different stages, attaining a requirement of this model [22]. Previous studies demonstrated that the SoC algorithm have a good-to-excellent reliability [23]. Moreover, the construct validity of the SoC algorithm is broadly supported in literature [24,25].

Influence of seasonality on PA is another aspect to be considered. As the level and the motivation to engage in PA tend to increase in spring and summer [26], and the present study was undertaken in spring, the prevalence of individuals who initiated PA or who intend to do so may be overestimated. In Southern Brazil, seasons are well defined, and during spring it is common to find a growing interest towards PA, and demand for physical fitness centers is often higher. However, this effect consists of a non-differential error, because all individuals were interviewed in the same season.

Interpretation of the associations between the SoC for PA and the covariates should consider the study design. As all variables were measured simultaneously, their interrelationship does not necessarily reflect a causal association. Nevertheless, the present study was designed to determine characteristics of individuals in each stage of change. Further, the extrapolation of the results to other populations should be made with caution. More studies from different areas of Brazil and from other developing countries are needed to know if the data can be really generalized to other settings.

The comparison of the findings is sometimes difficult, because concept of the stages, assessment formats and definition of PA significantly vary between studies. The most divergent concept is related to the preparation stage. Some studies define this stage as engaging in PA but not on a regular basis, while others consider the intention to engage in PA within the next 30 days. Because the individual may be irregularly active with no intention to become regularly active, characterizing the precontemplators, in the present study, the authors chose the second definition. Moreover, some studies include another stage (relapse), which presumes that individuals regress to an earlier stage in their attempts to change behavior. But, as it is the rule, rather than the exception [27], this stage was not considered in the present study.

The assessment format used was a yes/no, instead of a five-choice format, although both formats are appropriate to classify the individuals in the stages [21]. Concerning the definition of regular PA, some studies do not specify the criteria to define it, or simply consider the intention to increase PA level, while others use the recommendation that encourages 30 accumulated minutes of PA over the day [28]. The criterion of regular PA adopted in this study was in accordance to the recommendations of the American College of Sports Medicine (ACSM) to maintain cardiorespiratory fitness and body composition in adults [29]. Furthermore, it was the most frequent criterion adopted by studies included in a meta-analysis about this theme [20]. Besides, lifestyle PA may overestimate the prevalence of post-adoption stages and put fewer individuals into the pre-adoption stages [30].

Overall, 29.2% of the adults interviewed met the criterion to be physically active. This prevalence was higher than reported in other surveys conducted in Brazil [2,31], suggesting that the SoC algorithm overestimates the prevalence of PA, as corroborated by Marshall and Biddle [20]. This may be due to the more rigorous criteria adopted for PA in other investigations, and to the fact that more details are collected from PA questionnaires. Even so, many authors apply the SoC algorithm to evaluate the prevalence of PA because of its ease of administration and scoring.

Compared to a similar research, carried out among a representative sample of adults from the US, the prevalence of the precontemplation and the maintenance stage in the present study was respectively twice and one-half than that obtained by Laforge et al. [32]. However, the prevalence of the contemplation, preparation and action stages were very similar among these two researches. In general, the prevalence of precontemplation obtained in this study was markedly higher than in most studies in the literature, while the proportion of individuals in the maintenance stage was lower than that found in most investigations related to the SoC for PA in adults.

Regarding covariates, the association between the SoC for PA and sex was consistent with other investigations in which women were more frequently in the contemplation, preparation and action stages and less frequently in the maintenance stage, with no differences in the precontemplation stage, compared to men [11]. This finding demonstrates that although women are less active than men, they are more likely to adopt PA.

Skin color (non-white) was associated only with the precontemplation stage. Another study conducted among women from various racial/ethnic groups found that a higher proportion of black women were in the precontemplation stage, compared to white women [30]. It suggests that, besides being more sedentary, black individuals are usually less motivated to engage in PA.

The differences between marital status and the SoC for PA has been shown in other studies, indicating that the prevalence of the precontemplation stage is higher for those individuals with a partner, while the prevalence of individuals in the maintenance stage is higher for those without a partner [33].

The association between age and the SoC for PA tends to be divergent. Most studies found that the proportion of older individuals are higher in the precontemplation stage and lower in the maintenance stage [33]. In the present study, age was positively associated with the precontemplation stage, and showed an inverse association with the other stages in the multinomial analysis.

Economic status, family income and education level demonstrated a similar trend towards the SoC, indicating that those in higher socioeconomic status are more likely to adopt and maintain PA. However, few studies have examined the distribution of these variables across the SoC for PA, hindering comparisons between studies.

With regard to smoking, other investigations found that the proportion of current smokers are higher in the precontemplation stage and lower in the maintenance stage than for the non-smokers or former smokers [34]. In the present study, smokers were also less inclined to initiate and to maintain PA.

The results for BMI differ from previous researches that show a positive association with the precontemplation stage and a negative association with the maintenance stage [33]. In the present study, individuals with higher BMI were more likely to present intention to begin, and to have initiated PA in the last six months, suggesting that those above normal weight are more motivated to adopt PA. However, BMI was not associated with the maintenance of PA.

In relation to self-reported health status, the better the individuals perceived their own health, the more likely they were to maintain PA, even after adjusting for possible confounders. Only one research investigating the association of this variable with the SoC for PA was found, and showed consistent results with those of the present study [35]. Another investigation verified that the self-perceived quality of life increases across the SoC for PA [32].

A research investigating the predictors of PA adoption over four years in a community sample identified that the proportion of individuals who maintained PA was higher for those with higher income, more education, non-smokers, and those who had lower BMI [36].

The findings of the present study have several implications for research and practice. It appoints that the individuals in the contemplation and preparation stages are more similar to those individuals in the action and maintenance stages than to those in the precontemplation stage. Another interesting finding is that the variables associated with a certain behavior are not necessarily the same of those associated with the change of the behavior. For example, although men were more active than women, women were more likely to adopt and initiate PA than men. Adding, obese individual were more motivated to engage in regular PA compared to non-obese individuals.

Then, the studies that classify individuals only as physically active or inactive may hide some important information, because in the cluster of inactive individuals there may be some individuals who intend to engage in PA and some who have no intention to this. In the same way, among those who are active, there are some individuals that engaged in PA recently, and are more likely to drop out, and other that have already incorporated the practice of PA in their lifestyle.

Therefore, when programming effective interventions regarding PA, it is necessary to know not only the current level of PA, but also time of engagement, intention and other factors associated with the adoption of PA. Classifying individuals according to the readiness for PA is important because not all are in a stage in which they intend to make changes in their behaviors. Furthermore, interventions aimed at individuals who are motivated to adopt a physically active behavior may not produce an effect in a large proportion of the population.

There is strong evidence that stage-matched interventions may have broad applicability in increasing PA levels and promoting more effective use of resources, rather than action-oriented interventions [37]. Though, the SoC could be moderators of the relationship between intervention and behavior [38]. Moreover, the SoC algorithm can be easily used by physicians in counseling patients, since most people visit physicians each year, and they may positively influence patient behavior [39].

Although a sedentary individual may not immediately become a physically active person following an intervention, prompting individuals to begin considering an increase in their PA level is an important public health achievement. The singular progression to a further SoC for PA may be conducive to a more favorable health profile [40]. Thus, interventions that do not necessarily produce higher PA levels among precontemplators or contemplators, but do lead individuals to the preparation stage can also be considered successful [8].

Concluding the discussion, it is important to point out that motivating people to adopt and maintain PA over time constitutes a public health challenge. It seems that interventions must consider the SoC for PA to be successful. Other studies are needed to identify environmental factors associated with the SoC for PA and to investigate the variables that mediate behavior change related to PA.

## Conclusion

In summary, almost 40% of the adults from Southern Brazil are not engaged in PA and have no intention to do so in the next six months; about 30% were in the action or maintenance stage; and those intending to engage in PA (contemplation and preparation stages) represent the remaining 30%. The profile of those who are not engaged in PA but intend to do so is markedly more similar to those who are physically active than to those who are inactive and do not present intention to begin PA. Interventions to improve PA levels of the population should address mainly those people who are more resistant to adopt and to maintain PA, that are: the elderly, married, smokers and those with lower socioeconomic status.

## Competing interests

The author(s) declare that they have no competing interests.

## Authors' contributions

SCD coordinated the fieldwork and performed the data analysis. DPG and MRD supervised all phases of the study. All authors were responsible for the writing and approval of the final version of the manuscript.

## Supplementary Material

Additional File 1 Stages of change for physical activity algorithm. Instrument proposed to evaluate the stages of change for physical activity in adults. English version of the instrument applied in this study to measure the stages of change for physical activity. Click here for file
