# Supplementary material for: Stages of change for physical activity in adults from Southern Brazil: a population-based survey
Source: Int J Behav Nutr Phys Act. 2007 Jun 8;4:25. doi: 10.1186/1479-5868-4-25 (PMC1904456; doi:10.1186/1479-5868-4-25)
Supplement: Additional File 1 — Stages of change for physical activity algorithm. Instrument proposed to evaluate the stages of change for physical activity in adults. English version of the instrument applied in this study to measure the stages of change for physical activity. [file 1479-5868-4-25-S1.pdf]

Instrument proposed to evaluate the stages of change for physical activity in adults

Let's talk about physical activities, such as walking, exercises and sports, performed on a regular basis, and that increase your breathing or heart rate.

1) Do you currently engage in regular physical activity, that is, 3 days per week for at least 20 minutes each time?
   (0) No → go to the next question
   (1) Yes → go to question 4
2) Do you intend to engage in regular physical activity in the next 6 months?
   (0) No → finish the questionnaire
   (1) Yes → go to the next question
3) Do you intend to engage in regular physical activity in the next 30 days?
   (0) No → finish the questionnaire
   (1) Yes → finish the questionnaire
4) Have you been engaged in regular physical activity for the past 6 months or more?
   (0) No → finish the questionnaire
   (1) Yes → finish the questionnaire

<u>Staging algorithm:</u>

Precontemplation: question 1 = no; question 2 = no.

Contemplation: question 1 = no; question 2 = yes; question 3 = no.

Preparation: question 1 = no; question 2 = yes; question 3 = yes.

Action: question 1 = yes; question 4 = no.

Maintenance: question 1 = yes; question 4 = yes.
